# THE EFFECT OF SOCIAL CAPITAL AND ETHNICITY ON THE WELL-BEING OF OLDER ADULTS IN HONG KONG

**DOI:** 10.1093/geroni/igae098.0625

**Published:** 2024-12-31

**Authors:** Daniel W L Lai, Zideng Huang, Alison Yu-ting Ou

**Affiliations:** Hong Kong Baptist University, Hong Kong, Hong Kong; Hong Kong Baptist University, Hong Kong, Hong Kong; Hong Kong Baptist University, Hong Kong, Hong Kong

## Abstract

Ethnic minority older adults in Chinese societies remain understudied despite the increasing diversity of the aging population. This study investigated the influence of social capital determinants on the quality of life of South Asians and Chinese residents in Hong Kong, highlighting the importance of understanding each ethnic group’s unique social dynamics and cultural contexts for effective interventions. A telephone survey was conducted with a total of 1,015 Chinese (800) and South Asian (215) older adults aged 55 and above, using the World Bank’s tool to measure social capital across six dimensions: social participation, support, connection, trust, cohesion, and reciprocity and the World Health Organization’s Quality of Life questionnaire to assess quality of life. Demographic variables such as gender, age, marital status, education, and ethnicity were also collected. Hierarchical regression analyses showed that social capital factors such as social support, connection, and reciprocity negatively influenced the quality of life for Chinese Hong Kong residents but positively for South Asians. Social trust and belonging showed no significant effect on Hong Kong residents’ quality of life but improved it for South Asians. Ethnic group was a consistent moderator, with South Asians generally reporting lower quality of life than Hong Kong residents. The interaction between social capital and ethnicity has a significant impact on quality of life, with certain aspects of social capital benefiting South Asians more than Hong Kong residents. This suggests that quality of life interventions should be tailored to the specific social capital dynamics and cultural contexts of each ethnic group.

